# Dopamine Receptors: Is It Possible to Become a Therapeutic Target for Depression?

**DOI:** 10.3389/fphar.2022.947785

**Published:** 2022-08-17

**Authors:** Fangyi Zhao, Ziqian Cheng, Jingjing Piao, Ranji Cui, Bingjin Li

**Affiliations:** ^1^ Jilin Provincial Key Laboratory on Molecular and Chemical Genetic, The Second Hospital of Jilin University, Changchun, China; ^2^ Engineering Laboratory for Screening of Antidepressant Drugs, Jilin Province Development and Reform Commission, Changchun, China

**Keywords:** dopamine receptors, pathogenesis of depression, neural circuits, signaling pathway, brain regions

## Abstract

Dopamine and its receptors are currently recognized targets for the treatment of several neuropsychiatric disorders, including Parkinson’s disease, schizophrenia, some drug use addictions, as well as depression. Dopamine receptors are widely distributed in various regions of the brain, but their role and exact contribution to neuropsychiatric diseases has not yet been thoroughly studied. Based on the types of dopamine receptors and their distribution in different brain regions, this paper reviews the current research status of the molecular, cellular and circuit mechanisms of dopamine and its receptors involved in depression. Multiple lines of investigation of these mechanisms provide a new future direction for understanding the etiology and treatment of depression and potential new targets for antidepressant treatments.

## GRAPHICAL ABSTRACT

The pathogenesis of depression cannot be separated from the involvement of various brain regions. Dopamine and its dopamine receptors are widely distributed in various regions of the brain, but the role and exact mechanisms of dopamine receptors in psychiatric disorders such as depression have not been thoroughly investigated. In this paper, we review the current status of research on the molecular pathways and mechanisms of dopamine receptor involvement in depression from the perspective of the types of dopamine receptors and their distribution in different regions of the brain, including the progress of research on the role of dopamine receptor D1-D2 heterodimers, which provides potential new targets for antidepressant drug therapy.
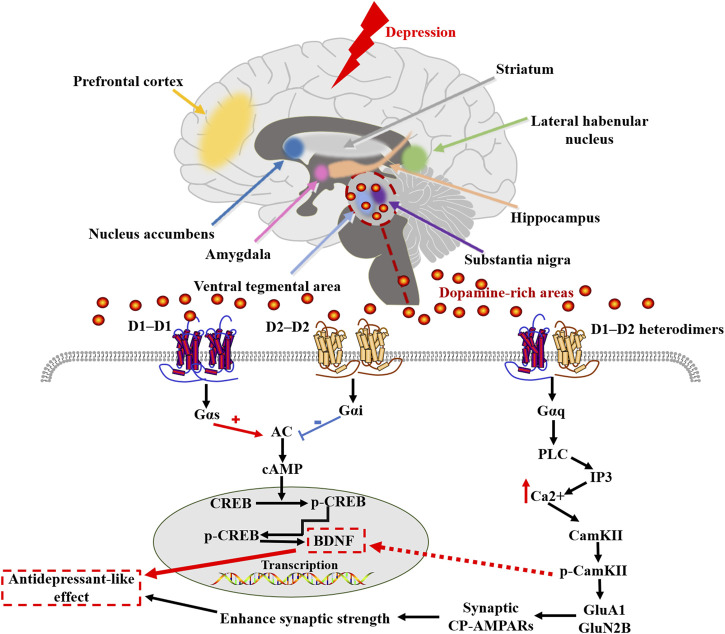

## Introduction

Depression is one of the most common chronic psychiatric disorders with a high morbidity and recurrence rate, which is mainly characterized by low mood, cognitive dysfunction, the inability to experience pleasure from normally rewarding stimuli (anhedonia), despair, sleep disturbance, etc. (Malhi and Mann, 2018; Rehm and Shield, 2019; Rice et al., 2019). According to the World Health Organization, by 2030, depression will be the leading cause of disability worldwide (Mathers and Loncar, 2006; Brhlikova et al., 2011). Depression is a public health problem that needs to be addressed urgently, but its etiology and pathophysiology remain to be fully understood despite many advances in the understanding of this disease (Fox and Lobo, 2019). Several hypotheses have been proposed to explain the causes of depression. These include the monoamine hypothesis (Schildkraut, 1965), the monoamine receptor hypothesis (Sulser et al., 1978; Charney et al., 1981), inflammation hypothesis (Normann and Cornelius, 1978), neural immunity hypothesis (Andreoli et al., 1989), neurotrophic factor hypothesis (Altar, 1999), hypothalamic-pituitary-adrenal (HPA) axis hypothesis (Kathol et al., 1989; Nestler et al., 2002), neurogenesis hypothesis (Malberg et al., 2000), neuronal and synaptic plasticity hypothesis (Schinder and Poo, 2000) as well as neural circuit hypothesis (López et al., 1999). Each hypothesis is related to specific cellular pathways and mechanisms of interaction between parts of the neural circuits underlying emotional, motivational, mnemonic, and cognitive deficits in depression (Peng et al., 2015). There is a certain degree of evidence for cellular and circuit level changes underlying each of these hypotheses, but many questions remain unanswered. The monoamine neurotransmitter hypothesis is the most widely studied hypothesis at present, which suggests that the occurrence of depression is related to reduced function of the monoamine neurotransmitters such as norepinephrine (NE) and serotonin (5-HT), and in more modern conceptions of the hypothesis, also dopamine (DA) (Nutt, 2008). Currently, the first-line drugs clinically used for the treatment of depression are all developed under the classical monoamine hypothesis, among which the most common antidepressants include tricyclic antidepressants (TCAs), selective 5-HT reuptake inhibitors (SSRIs), dual 5-HT and NE reuptake inhibitors (SNRIs), noradrenergic and specific serotonergic antidepressants (NaSSA), and other types (Schatzberg, 1998; Qin et al., 2014; McCormack, 2015; Blier, 2016). Nevertheless, these antidepressants generally have many disadvantages, such as slow onset, high rates of side effects, high recurrence rate, a high rate of interactions with other drugs, heterogeneous therapeutic responses, and other limitations (Peretti et al., 2000). These limitations suggest the need for additional research on the pathogenesis of depression to develop new antidepressant drugs based on a better understanding of the underlying mechanisms. One approach in this search for additional mechanisms is to study the receptors that specifically bind to the monoamine neurotransmitters.

In recent years, clinical research has shown that the function of the DA systems and DA receptors is involved in depression (David et al., 2020). This is not surprising because DA affects a variety of functions relevant to depression: emotion, perception, behavior, and motivation. However, recent research has shown that DA receptors and their heterodimers play a crucial role in the communication and connection of various neural circuits that may be involved in depression. Previously, DA receptors were thought to be primarily monomeric receptors, but now there is increasing evidence that multiple DA receptors can exist in the form of oligomers, forming homomeric and heteromeric receptor complexes, including ionotropic glutamate receptors (George et al., 2014; Guitart et al., 2014; Marsango et al., 2015). Some of these interactions may be regulated through signal transduction mechanisms, particularly through adenylate cyclase (AC) and cyclic adenosine monophosphate (cAMP) signaling (Beaulieu et al., 2015; Yeom et al., 2020). In recent studies, these complexes have been suggested to be a new direction for the study of the etiology of depression (Hasbi et al., 2020b; Hasbi et al., 2020c; Noori et al., 2020). Different DA receptor subtypes act on different neuronal pathways, which are likely to be the focus of research on the pathogenesis of depression (de Kwaasteniet et al., 2014).

## The Synthesis and Metabolism of Dopamine and Its Relationship With Depression

**FIGURE1:**
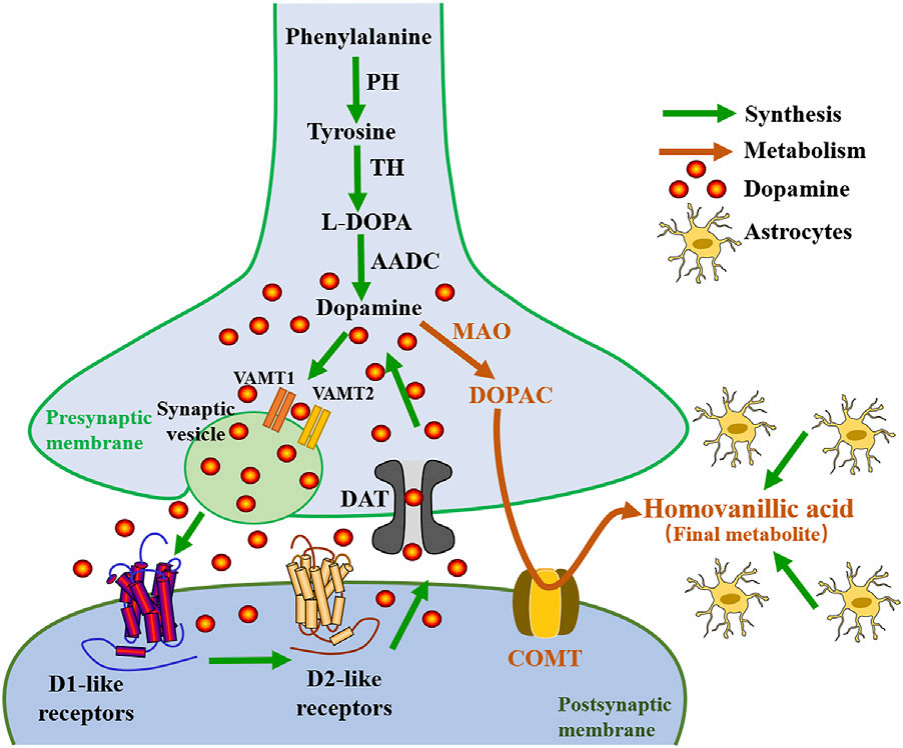
Diagram of the relationship between the synthesis and metabolism of dopamine. PH, phenylalanine hydroxylase; TH, tyrosine hydroxylase; l-DOPA, levo-dopa; AADC, aromatic L-amino acid decarboxylase; VMAT, vesicular monoamine transporter; LTP, Long-term potentiation; MAO, monoamine oxidase; DOPAC, 3,4-dihydroxyphenylacetic acid; COMT, catechol-o-methyltransferase.

Several animal models of depression are associated with decreased dopaminergic (DAergic) activity and anhedonia-like behavior. Anhedonia is thought to be a core feature of depression, and DA plays a key role in the perception of pleasure and reward, as well as motivated behavior, so it is important to discuss the normal roles of DA in behavior before exploring the relationship between DA receptors and depression. DA is the most abundant catecholamine neurotransmitter in the brain (Drozak and Bryla, 2005), which plays an important role in regulating rapid glutamate- and gamma-aminobutyric acid (GABA) -mediated neurotransmission in many brain regions, and is involved in many physiological and behavioral processes, including aspects of reward valuation and motivation, motor control and behavioral selection, attention and certain aspects of cognition, and some types of hormone secretion (Gainetdinov et al., 2002). There are five DAergic receptor subtypes in the G protein-coupled receptor (GPCR) superfamily (D1R, D2R, D3R, D4R, and D5R) (Gurevich et al., 2016). DA is synthesized directly from tyrosine by the enzyme tyrosine hydroxylase (TH), or indirectly from the essential amino acid phenylalanine (Franco et al., 2021), which is transformed into tyrosine by phenylalanine hydroxylase (PH) (Klein et al., 2019). As shown in Figure 1, when tyrosine enters the neuron, it is transformed into L-3,4-dihydroxyphenyl-L-alanine (l-DOPA) catalyzed by TH in the cytoplasm. l-DOPA is absorbed by large neutral amino-acid (LNAA) transporters and decarboxylated to DA by aromatic L-amino acid decarboxylase (AADC) present in neurons and glial cells. Studies have shown that l-DOPA plays a neuroprotective role on DAergic neurons through astrocytes (Asanuma and Miyazaki, 2016). As a metabolic precursor of DA, l-DOPA plays an important role in DAergic neurotransmission. Acute l-DOPA treatment enhances the transmission of DA in the substantia nigra and is one of the standard treatments for Parkinson’s disease (Cao et al., 2020). In addition, clinical studies have shown that l-DOPA improves the cognitive processing and gait speeds of elderly patients with depression (Rutherford et al., 2019). These improvements were associated with reduced binding of labeled raclopride in selected striatal subregions, indicative of increased DAergic neurotransmission. DA and glutamate released by midbrain DA neurons have different properties, which are reflected in different synaptic vesicle mechanisms (Silm et al., 2019). Vesicular monoamine transporters (VMAT) mediate the packaging and storage of the monoamines (5-HT, DA, histamine, adrenaline, and NE) (Yaffe et al., 2018). VMAT is responsible for the transport of cytoplasmic monoamines into synaptic vesicles for storage and subsequent extracellular release in the CNS (central nervous system) (Wimalasena, 2011). VMAT is responsible for the packaging and transport of neurotransmitter molecules into presynaptic storage vesicles prior to release into the synaptic cleft when an action potential or other signal leads to increased synaptic calcium levels (Gantz et al., 2015). Two closely related VMATs, VMAT-1 and VMAT-2 have been cloned, expressed, and characterized, and both have distinct pharmacological properties and tissue distribution characteristics (Wimalasena, 2011).

VMAT-1 is expressed preferentially in neuroendocrine cells and the peripheral nervous system, while VMAT-2 is mainly expressed in the CNS (Wimalasena, 2011). Although most research on DAergic mechanisms in the brain has focused on VMAT-2, a recent study by Lohoff et al. suggests that significant changes in DAergic signaling in the frontal cortex occur in VMAT-1 null-mutant mice (Lohoff et al., 2019). This suggests that the role of VMAT-1 in CNS function may be underappreciated and that it may be involved in functions relevant to the pathogenesis and/or treatment of psychiatric disorders. Genetic variation in the VMAT-1 gene (*SLC18A1*) has been implicated in the activity of neural circuits associated with emotion, it plays an important role in brain structural changes in patients with depression (Vaht et al., 2016; Won et al., 2017). DA signaling and distribution are mainly regulated by VMAT-2 and DA transporter (DAT) proteins, which transport DA to synaptic vesicles and presynaptic terminals, respectively, and are regulated by complex processes such as phosphorylation and protein-protein interactions (German et al., 2015). Conditional deletion of VMAT-2 in astrocytes leads to loss of prefrontal cortex (PFC) DA homeostasis, resulting in impaired synaptic transmission and plasticity as well as impaired executive function (Petrelli et al., 2020). Petrelli et al. concluded that the lack of VMAT-2-dependent DA stores in astrocytes causes an abnormal increase in mitochondrial enzyme monoamine oxidase B (MAOB) and the plasma membrane organic cation transporter 3 (OCT3) activity, which leads to a decrease in extracellular DA levels (Petrelli et al., 2020). Clinical pharmacological studies have shown that the uptake of DA by monoaminergic neurons mediated by VMAT-2 can prevent the oxidation of DA, and the overexpression of VMAT-2 may provide a potential target for neuroprotective therapy in various psychiatric diseases (Segura et al., 2019). DAT is a plasma membrane glycoprotein selectively expressed in the presynaptic membrane of central DAergic neurons (Mortensen and Amara, 2003). It belongs to the Na+ CI− dependent membrane transporter gene family and is most densely distributed in the basal ganglia (Wang et al., 2015). Accurate regulation of synaptic DA levels by DAT ensures the phasic nature of the DA signal, which underlies the ability of DA to encode reward prediction errors. The spatial and temporal strength of DA signaling is largely dependent on the role of DAT, which regulates both extracellular and intracellular DA levels (Giros et al., 1996). Pharmacological changes in DAT function not only modulate DA reuptake, but also induce rapid alterations in the plasmalemmal expression of the transporter (Kahlig and Galli, 2003). DAT is regulated by different presynaptic proteins, including DRD2 and DRD3 (acting as sensors of extracellular DA concentration, regulating the synthesis and release of DA), and abnormal DAT function is closely associated with several neurodegenerative diseases and psychiatric disorders (Rouge-Pont et al., 2002; Sokoloff et al., 2006). Research by Condon et al. (2019) shows that DAT is the primary regulator of DA short-term plasticity, controlling the balance between release-dependent and release-independent mechanisms. Some studies suggest that decreased DAT availability may be a hallmark of anhedonic depression, suggesting that DAT may serve as a specific therapeutic target for patients with high levels of anhedonia (Camardese et al., 2014). Degradation of DA occurs via two enzymatic processes catalyzed by MAO and cathecholamine O methyltransferase (COMT), which produces homovanillic acid (HVA, a primary DA metabolite) (Franco et al., 2021) (see Figure 1).

MAO is a mitochondrial enzyme that inactivates DA in the brain. It was concluded that the MAOB and COMT are mainly expressed in astrocytes (Cahoy et al., 2008; Petrelli et al., 2020). In fact, the HVA is made in glial cells (astrocytes). Astrocytes can coordinate neural development by orchestrating synapse formation and function, which may be closely related to the pathogenesis of neurodevelopmental abnormalities common in psychiatric disorders (Chung et al., 2015). Reduced COMT activity in the PFC predicts a decrease in midbrain DA synthesis (Meyer-Lindenberg et al., 2005). COMT variants that alter DA function also affect prefrontal cortical connectivity, and these differences are associated with depression (Na et al., 2018). The COMT Val158Met polymorphism affects levels of DA, which plays an important role in depression (Camardese et al., 2014; Otsuka et al., 2019). Inoue et al. have shown that transmembrane protein 132D (TMEM132D), COMT, and GABA receptor alpha 6 subunits (GABR_A6_) genotypes are associated with emotional processing in the cingulate, frontal cortex, and hippocampus in panic disorder and major depressive disorder (MDD) (Papaleonidopoulos et al., 2018). Results have shown that the levels of HVA in the cerebrospinal fluid (CSF), are decreased in patients with depression (Reddy et al., 1992; Saloner et al., 2020). Antidepressant treatments reversed DAergic hypoactivity and anhedonia-like behavior, as well as increased HVA levels in CSF (Horikoshi et al., 2019), which suggests an important role of DA in the pathophysiology of depression.

## Classification and Distribution of Dopamine Receptors and Their Signaling Pathways

Based on their ligand recognition properties and their effect on cAMP, DA receptors were initially divided into two pharmacological families: D1-like receptors and D2-like receptors. D1-like receptors are coupled to Gs and Golf proteins, whose binding activates adenylate cyclase (AC), increasing the activation of the cAMP/PKA cascade response, and intracellular events resulting modification of cortico-striatal glutamatergic synapses (Beninger and Miller, 1998). Signaling cascades activated by D1-like receptors can also have long-term effects on cellular function by regulating transcription. For example, D1-like receptor agonists increase cAMP levels and the phosphorylation of the cAMP-response element binding protein (CREB) at Ser133, which subsequently regulates the transcription of many genes that are important for a variety of psychiatric disorders (Zhang et al., 2016). Related studies have suggested that the behavioral effects of some D1 agonists are not related to cAMP/PKA signaling, but rather involve non-cAMP-mediated signaling, including phospholipase C (PLC)-mediated calcium elevation (O'Sullivan et al., 2004). SKF-83959 is a highly D1-biased ligand with a full agonistic effect (*via* Gαq) on D1-mediated activation of PLC signaling and an antagonistic effect on D1-mediated AC signaling (O'Sullivan et al., 2004). In contrast, D2-like receptors are coupled to Go and Gi proteins, which are involved in the inactivation of AC, resulting in a decrease in cytosolic cAMP levels (Beaulieu and Gainetdinov, 2011; Alexander et al., 2019). Binding of DA to D2-like receptors inhibit the cAMP/PKA signaling pathway ultimately affecting the CREB phosphorylation. Later, five DA receptor subtypes were cloned by molecular biology techniques: the D1-like receptors included the D1R and D5R subtypes, while the D2-like receptors included the D2R, D3R, and DRD4 subtypes (Table 1) (Undieh, 2010). There are two splice variants of the DRD2 gene that result in receptors of different lengths (number of amino acids), we were termed DRD2L (long) and DRD2S (short)]. DA receptors are mainly distributed in the CNS and peripheral nervous system. Among them, D1R and D2R are the most abundant subtypes in the CNS (Wang et al., 2008). D1-like receptors are mainly present postsynaptically, whereas D2-like receptors are present in postsynaptic DAergic target neurons and also act presynaptically as autoreceptors on DA neurons. The activity of DA receptors is extremely complex and is regulated by a variety of factors in different brain regions, including the ventral tegmental area (VTA), nucleus accumbens (NAc), Substantia nigra, PFC, hippocampus, amygdala, striatum, and lateral habenular nucleus (LHb), and ventral pallidum (VP). The localization of D1-like and D2-like receptors is different.

**TABLE1:** Classification, function, and localization of dopamine receptors in the brain as well as the relevant signaling pathway.

	Dopamine receptor subtypes				
	D1-like receptor		D2-like receptor		
	D1 receptor	D5 receptor	D2 receptor	D3 receptor	D4 receptor
Second messenger effect	Increase AC	Increase AC	Decrease AC	Decrease AC	Decrease AC
Cognate G protein	Gαs/olf	Gαs/olf	Gαi/o	Gαi/o	Gαi/o
cAMP production	Stimulate	Stimulate	Inhibit	Inhibit	Inhibit
Localization	Striatum	Ventral tegmental area	Nucleus accumbens	Olfactory nodules	Prefrontal cortex
	Nucleus accumbens	Striatum	Olfactory nodules	Nucleus accumbens	Anterior motor cortex
	Prefrontal cortex	Thalamus	Striatum	Striatum	Cingulate cortex
	Substantia nigra	Hippocampus	Islands of Calleja	Amygdala	Substantia nigra
	Amygdala	Olfactory nodule	Substantia nigra	Hypothalamus	Hypothalamus
	Hippocampus	Substantia nigra	Ventral tegmental area	Islands of Calleja	Hippocampus
	Thalamus		Hippocampus	Ventral tegmental area	Caudate nucleus
			Pituitary	Basal ganglia	Nucleus accumbens
				Prefrontal cortex	Ventral tegmental area
Relevant pathway	cAMP/PKA signaling	cAMP/PKA signaling	cAMP/PKA signaling	cAMP/PKA signaling	cAMP/PKA signaling
	DARPP-32 signaling	DARPP-32 signaling	DARPP-32 signaling	DARPP-32 signaling	DARPP-32 signaling
	ERK signaling	ERK signaling	GSK-3β signaling	GSK-3β signaling	GSK-3β signaling
	MAPK signaling	MAPK signaling	PI3K/AKT signaling	PI3K/AKT signaling	PI3K/AKT signaling

Note: AC, Adenylyl cyclase; cAMP, cyclic adenosine monophosphate; PKA, protein kinase A; DARPP-32, dopamine- and cAMP-regulated neuronal phosphoprotein; ERK, Extracellular signal-regulated kinases; GSK-3β, Glycogen synthase kinase-3beta; MAPK, mitogen-activated protein kinases; PI3K, phosphoinositide 3-kinase; Akt, protein kinase B.

**FIGURE 2:**
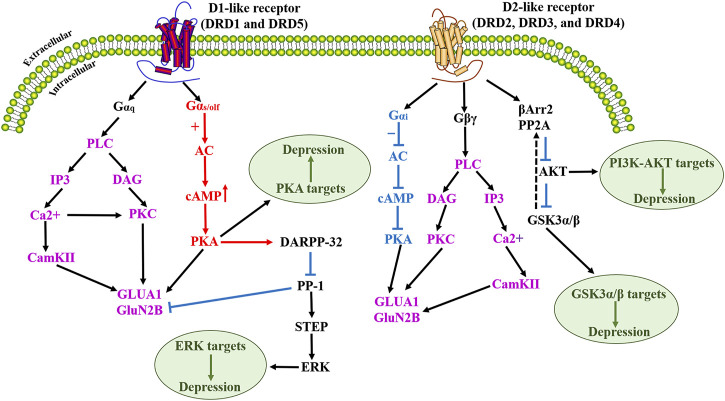
Schematic diagram of D1-like dopamine receptor and D2-like dopamine receptor activation signaling cascade. Upon activation of the dopamine D1-like receptor, activated PKA mediates the phosphorylation of DARPP-32, which acts as an effective inhibitor of PP-1, which in turn dephosphorylates another phosphatase STEP. Dopamine D1-like receptor activation mediates dopamine-dependent inhibitory cascades by increasing ERK phosphorylation by blocking the dephosphorylation of STEP. Numerous studies have demonstrated that modulation of the cAMP/PKA signaling pathway can improve depression. D2-like receptor activation promotes phosphorylation/activation of Akt and phosphorylation/inactivation of its substrate GSK-3β. The physiological significance of this D2 receptor-activated Akt/GSK3 signaling has been extensively discussed in terms of neuroprotection against oxidative stress in depression. In addition, numerous studies have also shown that the PI3K/Akt pathway has an integral role in the treatment of depression. PLC, Phospholipase C; IP3, Inositol triphosphate; CamKII, calmodulin-dependent protein kinase II; DAG, diacylglycerol; PKC, protein kinase C; GluA1, glutamate A1; GluN2B, Glutamate Receptor Ionotropic, NMDA 2B; AC, Adenylyl cyclase; cAMP, cyclic adenosine monophosphate; PKA, Protein Kinase A; DARPP-32, Dopamine- and cAMP-regulated phosphoprotein; PP-1, protein phosphatase-1; STEP, striatal-enriched tyrosine phosphatase; ERK, extracellular-signal-regulated kinases; βArr2, β-arrestin-2; PP2A, protein phosphatase 2A; Akt, protein kinase B; PI3K, phosphatidylinositol-3; GSK3, glycogen synthase kinase 3.

D1-like receptors are highly expressed in the striatum, NAc, substantia nigra, olfactory bulb, amygdala, and PFC, with lower levels of expression in the hippocampus, cerebellum, thalamus, and hypothalamus (Beaulieu et al., 2007; Dunlop and Nemeroff, 2007). In NAc, plasticity-related signaling of Ca^2+^/calmodulin-dependent protein kinase II (CamKII) and adenosine A2A receptors (A2ARs) are required for discrimination learning (Iino et al., 2020). Recent evidence suggests that all of these G-protein-mediated signaling cascades converge on, the phosphorylation of two ionotropic glutamate receptor subunits, GluA1 and GluN2B, which play a key role in glutamatergic transmission (Koutsokera et al., 2014). D1-like receptors are expressed in striatal GABAergic medium spiny neurons (MSNs) that project to the medial globus pallidus and the substantia nigra reticulata (SNr) (i.e., the direct nigrostriatal pathway), while DRD2 is expressed on the MSNs that project to the lateral globus pallidus (i.e., the indirect pathway). D1-like receptors influence the function of multiple voltage-gated ion channels, as well as N-Methyl-D-Aspartate (NMDA) and GABA_A_ receptors, by directly or indirectly acting on DARPP-32, the MAPK signaling pathway (such as ERK, JNK, P38), and other kinases and phosphatases (Chen et al., 2004). DRD2 receptor is mainly distributed in the hippocampus, striatum, thalamus, pituitary olfactory nodule, substantia nigra, and VTA (Missale et al., 1998). It has become increasingly clear that D2R acts through protein kinase B (Akt)-GSK-3 (glycogen synthase kinase 3) signaling cascade, and this signaling pathway involves the multifunctional scaffold protein β-arrestin2 (βArr2), which plays a role in GPCR desensitization (Beaulieu et al., 2007). The expression of D3R is relatively low in the central nervous system and is mainly distributed in the limbic system, including the NAc shell and olfactory tubercle (Missale et al., 1998). It also has a lower level of expression in other portions of the striatum, basal ganglia, the NAc core, islands of Calleja, substantia nigra, the VTA, hippocampus, septum, and various cortical regions (Le Moine and Bloch, 1996; Gurevich et al., 2016; Solís et al., 2017). DRD4 receptor is expressed at low levels in the basal ganglia and high expression in the striatum, frontal cortex, medulla, amygdala, hypothalamus, midbrain, and islands of Calleja, however, these levels are much lower than other DA receptors. DRD5 receptor also has lower levels of expression in other brain regions, including the PFC, anterior motor cortex, cingulate cortex, substantia nigra, hypothalamus, and hippocampus (Dunlop and Nemeroff, 2007). DRD5 also has a low level of expression overall, but this does include MSNs of the caudate nucleus and the VTA (Hernández-Echeagaray et al., 2007). However, the contribution of these receptors to circuit-level functional connections between brain regions remains poorly understood. Previous studies have shown that several subtypes of DA receptors may colocalize on some cells, but the receptors are largely segregated. The signaling pathway diagram for D1-like receptors and D2-like receptors is summarized in Figure 2, and a summary of all 5 DA receptors is given in Table 1.

The ultimate actions of DA receptor stimulation are also affected by dimerization. DA D1-D2 heterodimers are expressed in key cerebral cortical and subcortical regions in all species, and the differences in their expression in the striatum of different species suggest an evolutionary role of D1-D2 heterodimers in higher CNS function (Hasbi et al., 2020c). D1-D2 receptor heterodimers in subsets of neurons were first found in the rat striatum and are coupled with Gαq proteins to regulate intracellular calcium signaling (Perreault et al., 2012a), directly linking DA and calcium signaling (Perreault et al., 2012a; Perreault et al., 2014). Related studies have shown that the expression of D1-D2 receptor heterodimers in the striatum of juvenile rats is lower than in adult rats, and as result juvenile rats are less sensitive to D1-D2 receptor combined stimulation (Perreault et al., 2012b). This suggests that there may be significant age-dependent neurotransmission differences in the D1-D2 receptor heteromeric pathway combined with an *in situ* proximity ligation assay (PLA) technique with different neuronal markers to characterize the neurons expressing D1-D2 receptor heterodimers in the striatum (including the caudate nucleus, the putamen, and the NAc core and shell of the), finding heterodimers in all striatal regions and projection neurons of the direct and indirect basal ganglia pathways (Rico et al., 2017).

D1-D2 heterodimers induce calcium release via a Gαq-dependent pathway, distinct from G_s_/G_olf-_ or G_i_/G_o-_ dependent pathways activated by the D1 receptor or D2 receptor independently (Rashid et al., 2007; Hasbi et al., 2009). The increase in intracellular calcium content is rapid and transient, independent of extracellular calcium influx, and involves activation of Gq protein and phospholipase C (PLC) (Hasbi et al., 2009). D1-D2 heterodimers trigger calcium signaling by activating Gαq and PLC, leading to the activation of calmodulin kinase II-α (CaMKIIα) (Hasbi et al., 2009; Ng et al., 2010; Perreault et al., 2012a). Specific activation of D1-D2 receptor heterodimers in striatal neurons and the cellular co-expression of DRD1 and DRD2 leads to the intracellular release of calcium from stores sensitive to activation of inositol triphosphate receptors (IP3-R) (So et al., 2005). This calcium signaling results in an increased form of phosphorylation-activated form of CaMKIIα in striatal neurons and rat striatum (So et al., 2005). Phosphorylation of GluA1 and GluN2B plays a key role in the glutamatergic transmission and is regulated by the D1-D2 heterodimers signaling pathway. Expression of the GluA1 subunit of the AMPA receptor is associated with anhedonia. Studies have shown that mice lacking GluA1 (mice with *Gria1* knockout) show a reduction in licking cluster size, a measure of palatability of feeding behavior, and GluA1 is necessary for hedonic responding (Strickland et al., 2021).

**FIGURE 3:**
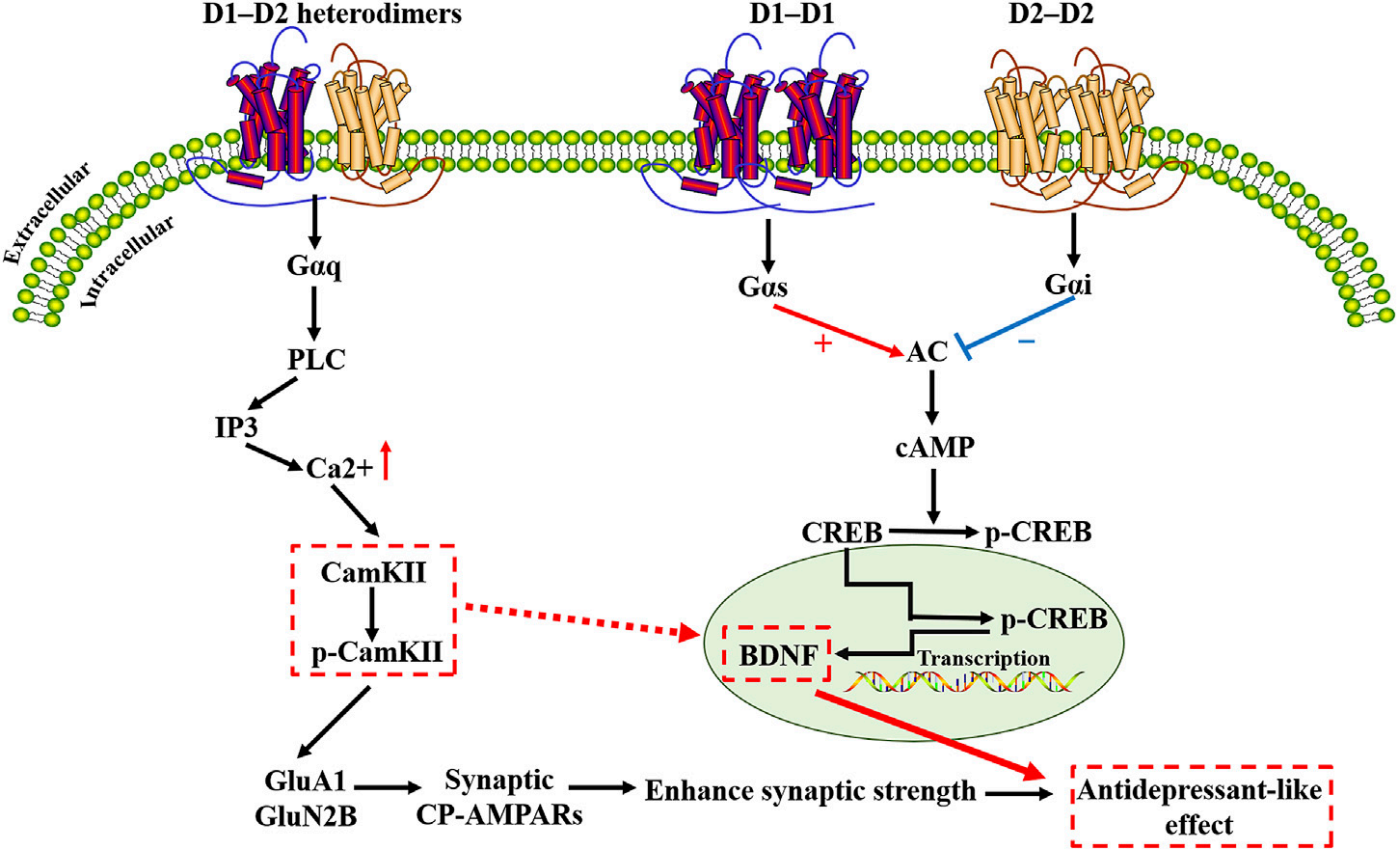
Schematic diagram of the dopamine receptor complex signaling cascade. D1-D2 heterodimers induce calcium release via a Gαq-dependent pathway, distinct from Gs/Golf- or Gi/Go- dependent pathways activated by D1 receptor heterodimers or D2 receptor heterodimers. D1-D2 heterodimers trigger calcium signaling by activating Gαq and PLC, leading to the activation of calmodulin kinase II-α(CaMKIIα). When calcium ions enter cells through various ion channels on the membrane, CaMKIIα is activated. Activation of CaMKIIα promotes its autophosphorylation, which binds to GluN2B and phosphorylates GluN2B at the S1303 site (p-GluN2B). Studies have shown that the interaction between GluN2B and CaMKIIα is significant for the synaptic CaMKIIα localization and activity. Phosphorylation of GluA1 and GluN2B plays a key role in the glutamatergic transmission and is regulated by the D1-D2 heterodimers signaling pathway. Inhibition of the recruitment, deletion, or activity of Ca(2+)-permeable AMPA receptors (CP-AMPARs) would interfere with long-term depression. CaMKIIα/BDNF/CREB-dependent neural plasticity pathways may be an important target for dopamine receptor complex research. D1R, dopamine D1-like receptor; D2R, dopamine D2-like receptor; PLC, Phospholipase C; IP3, Inositol triphosphate; CamKII, Calmodulin kinase II; p-CamKII, Phosphorylated CamKII; GluA1, glutamate A1; GluN2B, Glutamate Receptor Ionotropic, NMDA 2B; CP-AMPARs, calcium-permeable AMPA receptors; AC, Adenylyl cyclase; cAMP, cyclic adenosine monophosphate; CREB, cAMP-response element-binding protein; p-CREB, Phosphorylated-CREB; BDNF, Brain-Derived Neurotrophic Factor.

Information processing in the brain requires multiple forms of synaptic plasticity involving NMDA-type glutamate receptors (NMDAR) and AMPA-type glutamate receptors (AMPAR), including long-term potentiation (LTP) and long-term depression (LTD), and homeostatic scaling, potentially mediated by DA (Madadi Asl et al., 2018; Madadi Asl et al., 2022). PKA can anchor the scaffold protein AKAP150 to regulate GluA1 phosphorylation and plays a role in controlling Ca2+ -permeable AMPA receptor (CP-AMPAR) synaptic binding in NMDAR-dependent LTD (Sanderson et al., 2016). Inhibition of the recruitment, deletion, or activity of CP-AMPAR, would interfere with LTD, therefore, synaptic recruitment of CP-AMPAR is required to transiently increase NMDAR Ca (2+) signaling during LTD induction (Sanderson et al., 2016). On this basis, D1-D2 heterodimers-mediated signal transduction pathways may be thought to play an important role in other forms of synaptic plasticity as well, especially in LTP (Hasbi et al., 2009). Long-term synaptic plasticity is an essential form of brain plasticity. Inhibition of facilitated synaptic transmission may impair the function and structure of brain circuits implicated in the pathophysiology of depression, and antidepressants may counteract these alterations (Holderbach et al., 2007). Relevant findings suggest that activation of the D1-receptor complex, raises intracellular levels of cAMP, while the D2-receptor complex, inhibits intracellular levels of cAMP. The cAMP-response element binding protein (CREB) activates protein kinases in different DA receptors, such as protein kinase A (PKA), calmodulin-dependent protein kinase (CaMK) after phosphorylation at Ser133, and binds to the cAMP response element (CRE) of the target gene promoter (Santanavanich et al., 2005). This unique intracellular calcium signaling pathway links DA and brain-derived neurotrophic factor (BDNF) through a rapid increase in calcium signaling and CaMKIIα activation (Hasbi et al., 2009). Experimental studies have shown that basal levels of p-CAMKII, total CaMKII and BDNF are reduced in the CA1 region of the hippocampus of stressed rats (Alzoubi et al., 2013). BDNF, which is synthesized and released at glutamate nerve terminals, plays an important role in neuronal development by regulating protein synthesis and has been shown to increase the translation of hundreds of proteins isolated from synaptoneurosomes. In this review, we summarize recent studies on the etiology and pathogenesis of depression that involve different DA receptor mechanisms and different brain regions. The signaling pathway diagram summary of the DA receptor complex is shown in Figure 3.

## Dopamine Receptors and Depression

### Dopamine D1-Like Receptors and Depression

Positron emission tomography (PET) and functional magnetic resonance imaging (fMRI) can be used to study receptor binding potentials in the human brain *in vivo* (Hamilton et al., 2018). PET studies using radioligands for DRD1 have shown some promise as a means of researching the DA system in psychiatric diseases, ideally with higher selectivity radioligands, so that DRD1 can be evaluated as a candidate biomarker for disease and ultimately for treatment (Cervenka, 2019; Stenkrona et al., 2019; Yokokura et al., 2020). Currently, mice lacking DA D1 receptors are widely used to study the involvement of DA receptors D1-like class (D1 and D5) in motor and cortical striatal LTD and LTP, and endogenous DA stimulation of different subtypes of striatal neurons D1 and D5 receptors induces LTP and LTD, respectively (Kerr and Wickens, 2001; Rivera et al., 2002). Centonze et al. concluded that D1 and D5 receptors have different effects on the dependence of activity on both synaptic plasticity and spontaneous motor activity differently (Centonze et al., 2003). It has long been known that DRD1/DRD5 mechanisms regulate long-term plasticity and memory in the hippocampus (Hansen and Manahan-Vaughan, 2014). The mossy fiber (MF) synapse in the hippocampal CA3 region plays an important role in the molecular mechanisms of synaptic plasticity. Hagena et al. suggest that D1/D5 receptors are critical in regulating synaptic plasticity in MF-CA3 synapses, especially as a modulator of candidate processes for long-term memory (Hagena and Manahan-Vaughan, 2016). Lazenka et al. have shown that DA D1 receptor signal transduction is involved in behavioral pain-related depression in rats, suggesting that indirect and/or direct D1 receptor agonists might alleviate pain-related behavioral depression (Lazenka et al., 2017). In addition, Desormeaux et al. showed that modulation of selective DA D1-like receptor agonist A77636 induced antidepressant-like effects in rats (Desormeaux et al., 2020). Quetiapine is an atypical antipsychotic that is effective in treating depression and anxiety disorders. Male BALB/c mice injected with quetiapine every other day and pretreated with the D1 receptor antagonist SKF-35866 in the following experiments found a significant increase in the preference to the quetiapine-paired chamber in mice treated with 120 mg/kg quetiapine, and this effect was blocked by pretreatment with SKF-35866, suggesting that the antidepressant-like effects of quetiapine may be modulated by D1 receptors (Althobaiti, 2021). Recent experimental studies have shown that activation of the DRD1 receptor and PKA is involved in the memory-improving effect of acute physical exercise (Ramires Lima et al., 2021).

A large number of studies have shown that the impaired function of the medial prefrontal cortex (mPFC) is involved in depression. Hare et al. used optogenetics to stimulate the pyramidal cells expressing DRD1 in mPFC and found that the activation of the pyramidal cells expressing DRD1 could produce rapid and long-lasting antidepressant and anti-anxiety responses (Hare et al., 2019). The application of optogenetics techniques has made it possible to perform a more precise anatomical and cellular dissection of the role of specific DA receptors in DA-related functions (Beaulieu et al., 2015). Numa et al. showed that downregulation of D1R in mPFC reduces c-Fos expression in the interstitial nucleus of the posterior limb of the anterior commissure (IPAC) induced by social defeat stress. However, contrary to the above findings, Fedotova et al. showed that the D1 receptor antagonist SCH-23390 produced antidepressant-like effects in ovariectomized rats, where repeated administration of SCH-23390 greatly enhanced the antidepressant-like effects (Fedotova and Ordyan, 2011). However, the D1 receptor agonist SKF-38393 failed to alter depressi-like behavior in ovariectomized rats in FST but blocked the antidepressant-like effects of 17β-estradiol (17β-E2) (Fedotova and Ordyan, 2011). This suggests that D1 receptors may be activated by subthreshold social defeat stress in the mPFC. However, the specific mechanisms by which D1 receptor agonists and D1 receptor antagonists affect DAergic properties need to be further investigated.

### Dopamine D2-Like Receptors and Depression

With the development of novel radioligands, *in vivo* imaging can provide a new perspective on the pathophysiology of depression. An approach using PET has suggested that deep brain stimulation (DBS) of the medial forebrain bundle (MFB) partially reverses depression-like phenotypes associated with DRD2 blockade (Thiele et al., 2020). This effect appears to be related to increased levels of both DRD2 and DRD1. A growing body of evidence suggests that the direct D2-like receptor agonist pramipexole has antidepressant effects, particularly in electroconvulsive treatment (ECT) resistant depression (Gauthier et al., 2017) or in patients with deficits in baseline reward processing (Whitton et al., 2020). The effect of traumatic brain injury (TBI) on DA receptor binding was examined in patients with post-injury MDD (TBI-MDD) and patients without post-injury MDD (TBI-NON), as well as non-TBI control patients (Jolly et al., 2019). [11C]PHNO PET imaging was used to assess DRD2/DRD3 binding ratios (Le Foll et al., 2016). TBI was associated with reduced binding ratios overall, and these values were even lower in MDD patients, although this difference was not statistically significant. Given the small number of subjects, the finding is worthy of adding to determine whether DAergic mechanisms may be involved in post-traumatic depression. Fatima et al. (2020) suggested that selective D2 receptor agonists Ropinirole (ROPI) alleviate depression by upregulating tyrosine hydroxylase and increasing neurogenesis in the hippocampal region of prenatally stressed rats. Papp et al. (2019) found that both Wistar and Wistar-Kyoto rats (which have been validated as an animal model for treatment-resistant depression) exposed to chronic mild stress had reduced sucrose intake and impaired memory consolidation. Chronic treatment with serotonin and norepinephrine reuptake inhibitor venlafaxine reversed these effects in Wistar rats, while DBS reversed depression-like effects in Wistar-Kyoto rats (Papp et al., 2019). Venlafaxine reversed the effect of the DRD2 agonist L-742,626 on memory consolidation in unstressed, but not stressed, Wistar rats, while in Wistar-Kyoto rats, DBS reversed the effects of L-742,626, or the DRD3 agonist 7-hydroxy-N, N-di-N-propyl-2-aminotetralin (7-OH-DPAT) in both the stressed and unstressed rats (Papp et al., 2019). These results suggest that the effect of stress on memory consolidation impairment in rats can involve both DRD2 and DRD3 receptors in the ventral medial prefrontal cortex and that DBS effects on depressive symptoms may act in part through effects on DA function. The use of tractography for more refined deep brain stimulation electrode targeting and closed-loop deep brain stimulation approaches are the future trends in the treatment of depression (Dougherty, 2018).

A study in DRD3−/− mice demonstrated that the elimination of DRD3 receptors induces chronic depressive symptoms (Moraga-Amaro et al., 2014). Moreover, DRD3 expression and function are down-regulated during stress and depression, and antidepressant therapy can reverse these changes, suggesting that enhanced DAergic neurotransmission mediated by DRD3 down-regulation is involved in the adaptive changes underlying antidepressant activity. BDNF regulates the expression of DRD3 in certain brain regions, and the induction of BDNF by antidepressant therapy is related to behavioral outcomes (Leggio et al., 2013). Wang et al. (2020) used DRD3 KO mice to further demonstrate that DRD3 deficiency-induced depressive-like behavior involves neuroinflammation in mesolimbic brain regions, which helps us to understand DRD3 KO-induced depressive-like behavior and provides potential molecular and cellular targets for the treatment of depressive phenotypes. Experimental studies suggest that exposure to neonatal maternal separation (MS) and chronic mild stress (CMS) in adulthood completely inhibits reward-induced intra-NAc DA release, which is a useful indicator of depression severity and various therapeutic efficacy (Minami et al., 2017). Inflammation plays an important role in the pathophysiology of depression, and the peripheral administration of lipopolysaccharide (LPS) is one of the most common models of inflammation-induced depression. LPS results in a significant decrease in DRD3 in the VTA, mPFC, and NAc, key structures within the mesolimbic DAergic system (Wang J. et al., 2018). Pre-treatment stratification in depressed patients may be beneficial by taking into account the role of multiple anti-inflammatory agents in depression. DRD4 is also associated with the pathophysiology of several psychiatric disorders characterized by cognitive deficits, including depression (Rondou et al., 2010; Navakkode et al., 2017). Previous studies have shown increased expression of DRD4 in the basal amygdala of depressed patients compared to control subjects, and *in vivo* imaging studies of depressed patients show results consistent with those *post mortem* findings (Xiang et al., 2008). DRD4 appears to have a homeostatic role on synapses that stabilizes neural network activity. Navakkode et al. demonstrated that DRD4 plays a bidirectional role in the CA1 region of the hippocampus (Navakkode et al., 2017). Blocking DRD4 affects late-LTP and transforms early-LTP into late LTP (Navakkode et al., 2017). This enhanced LTP was dependent on protein synthesis, NMDAR activation, and CaMKII phosphorylation, as well as GABA_A_-receptor, mediated mechanisms (Navakkode et al., 2017).

### Dopamine D1-D2 Receptor Heterodimers and Depression

DA receptors are involved in homomeric and heteromeric complexes, which provide new targets for antidepressant drug discovery and are important for a deeper understanding of the complex physiological roles of these receptors in the brain (Perreault et al., 2014). It is necessary to further understand the role of DA receptor changes in specific brain regions of depressed patients and to determine the specific DA receptor mechanisms and other molecular complexes that underlie these functional changes that lead to depressive symptomatology. This will help to elucidate the pathophysiology of depression and aid in the development of new drugs with greater efficacy and fewer side effects. Recently, an increasing number of articles have highlighted the ability of both DRD1 and DRD2 to form heterodimers, and a growing body of evidence has linked D1-D2 heterodimers to drug addiction, Parkinson’s disease, schizophrenia, depression, and anhedonia (Shen et al., 2015; Hasbi et al., 2020b; Hasbi et al., 2020c; Noori et al., 2020). D1 and D2 receptors can form a heterodimeric complex that is present in a heterologous system and primary striatal neurons, as well as in the rodent brain *in vivo* (Perreault et al., 2014). However, most studies carried out to date stem from observations in heterologous systems and the biological significance of DA receptor heterodimers *in vivo* is only beginning to emerge. Recent data from *in situ* assessment of mRNA expression using RNA-FISH techniques revealed significant co-localization of DRD1 and DRD2 receptor mRNAs in the NAc, amygdala, piriform cortex, olfactory tubercle, claustrum, prelimbic cortex, and orbitofrontal cortex (Hasbi et al., 2020c). Perreault et al. (2014) showed that D1-D2 heterodimers may differentially regulate c-fos expression in a region-dependent manner either through its activation or through tonic inhibition of neuronal activity.

Studies have demonstrated that D1-D2 receptor heterodimers are upregulated in the postmortem brain of patients with depression and have identified an interfering peptide that disrupts D1-D2 receptor interactions (Pei et al., 2010). Pei et al. (2010) used the interfering peptide Tat-D2L_IL3-29-2_ to block the D1-D2 receptor heterodimers, significantly reducing immobility time in the forced swimming test without affecting locomotor performance and reducing escape failure in the learned helplessness tests in rats. This implies that the D1-D2 receptor heterodimers play an important role in the pathology of depression. Regulation of D1-D2 receptor heterodimers may be a novel pharmacological target for the treatment of depression and anxiety disorders, particularly addressing the high incidence of these conditions in females (Hasbi et al., 2020c). The coupling between DR1 and DR2 receptors in the brain is significantly increased in MDD patients (Hasbi et al., 2020c). A new study suggests that genetic interactions between DA receptor regulatory regions may influence the level of depressive symptoms through epistatic interactions between DRD1 and DRD2 regulatory elements that may affect D1-D2 heterodimers function (Corrales et al., 2016). Both DRD1 and DRD2 can form homomers and heterodimers, and the receptor configurations in the homomeric and heteromeric states appear to involve changes in their respective intracellular conformations, producing different G-protein coupling and subsequent activation of different signaling pathways (Hasbi et al., 2014). There are currently no selective antagonists targeting the D1-D2 heterodimers, but serial deletions and point mutations have been used to identify the amino acids involved in the interaction interface between the receptors. Residues in the DRD1 receptor located in the carboxylic tail interact with the DRD2 receptor to form D1-D2 receptor heterodimers (Hasbi et al., 2014). Interfering peptides block the formation of D1-D2 heterodimers and block the calcium signaling pathways activated by D1-D2 heterodimers, revealing a role of the D1-D2 complex in regulating behavioral despair *in vivo* (Hasbi et al., 2014). This interfering peptide may represent a new pharmacological tool to selectively disrupt GPCR heterodimers activity without affecting the function of the constituent receptors to elucidate the functional and behavioral consequences of D1-D2 heterodimers activity (Hasbi et al., 2014), and perhaps a potential therapeutic avenue for affecting heterodimer function in the absence of effects on the individual receptors. Identification of an interfering peptide that interferes with D1-D2 receptor heterodimers and has antidepressant-like effects may provide a new therapeutic strategy for the treatment of major depressive disorder (Pei et al., 2010; Duan et al., 2013; Brown and Liu, 2014).

## Circuit Mechanisms of Depression Related to Dopamine Receptors (Interesting New Targets)

**FIGURE 4:**
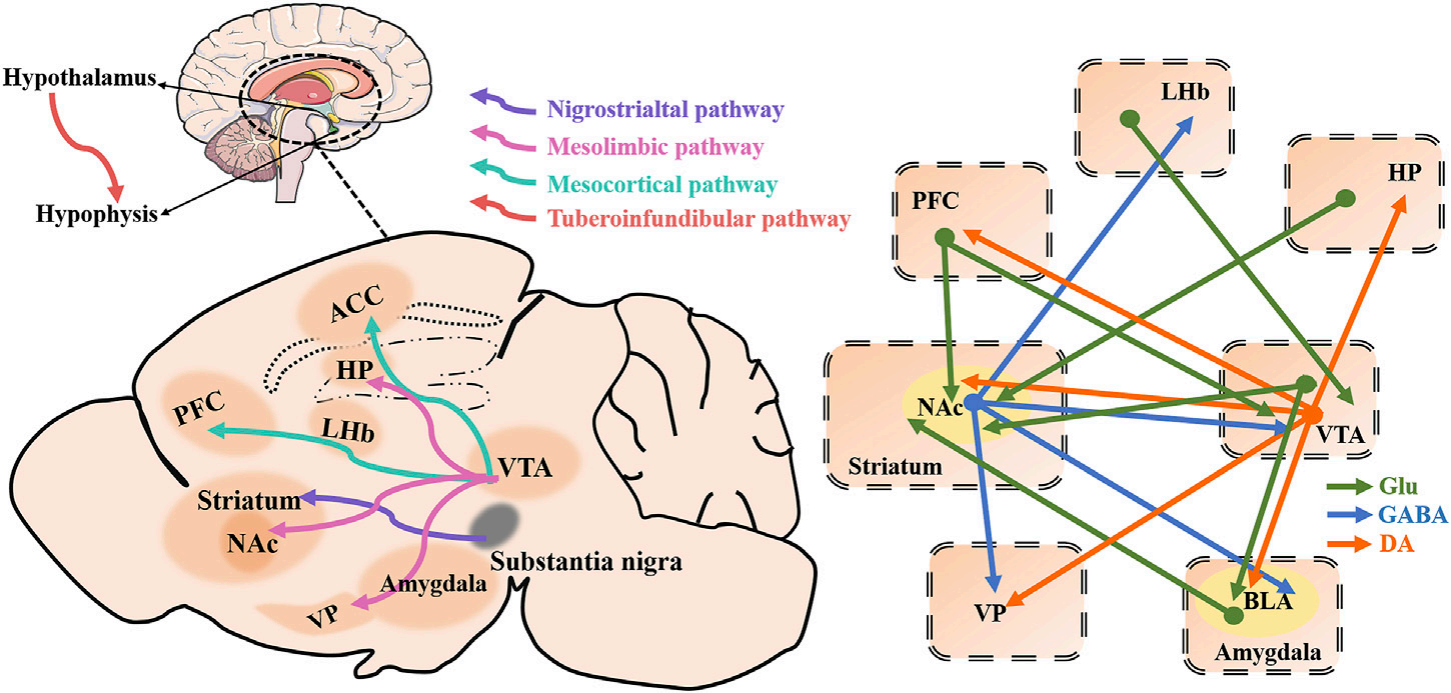
Four major brain dopaminergic pathways as well as depression-related brain regions and a schematic of brain circuitry implicated in depression. Dopaminergic (DAergic; orange) and glutamatergic (Gluergic; green) direct inputs converge on γ-aminobutyric acid (GABA)ergic (blue) neurons in the NAc to regulate behaviors of depression. The dopaminergic pathway from the VTA to the NAc plays a key role in reward-related behavior. Direct dopaminergic outputs of the VTA arise from the NAc, PFC, VP, BLA, and Hp, while the VTA, VP, BLA, and LHb receive NAc GABAergic inputs as well VTA, PFC, BLA, and Hp glutamatergic inputs. Besides, VTA receives PFC and LHb glutamatergic inputs. ACC, anterior cingulate cortex; HP, hippocampus; LHb, lateral habenula; PFC, prefrontal cortex; NAc, nucleus accumbens; VTA, ventral tegmental area; VP, ventral pallidum.

Four major brain DAergic pathways are involved in mammalian brain function: the nigrostriatal pathway (from cells in the A9 region), the mesolimbic and the mesocortical pathways (often collectively termed the mesocorticolimbic pathway, from cells in the A10 region, and the thalamic-tuberoinfundibular pathway (from cells in the A12 region) (Beaulieu and Gainetdinov, 2011). The nigrostriatal pathway projects from the substantia nigra pars compacta to the dorsal striatum (caudate and putamen) (Beaulieu and Gainetdinov, 2011). The mesocorticolimbic DAergic pathway is the most thoroughly studied DA pathway at present. The mesolimbic DA system, composed of DAergic projections from the VTA to the NAc, amygdala, hippocampus, and olfactory tubercle, plays a key role in reward-related learning, cognition, motivation, and decision-making processes (Polter et al., 2018; Heymann et al., 2020). The mesocortical pathway also originates in the VTA and projects into the frontal and temporal cortices, particularly the anterior cingulate cortex (ACC), entorhinal cortex, and PFC, which is thought to be important for attention and executive function (Dunlop and Nemeroff, 2007; Beaulieu and Gainetdinov, 2011). Some aspects of anterior pituitary function are also controlled by DAergic activity. The thalamic-tuberoinfundibular pathway originates from the arcuate nucleus of the hypothalamus (A12) and projects onto the median hypothalamic eminence (Ben-Jonathan and Hnasko, 2001). The details are shown in Figure 4.

### Ventral Tegmental Area

The VTA is a heterogeneous brain structure that plays a central role in reward-seeking and processing, learning, motivation, and neuropsychiatric disorders that involves alterations in these functions such as depression and addiction (Lüscher and Malenka, 2011; Cohen et al., 2012; Barker et al., 2016). Numerous studies have shown that DA neuronal activity of VTA plays a role in the pathophysiology of depressive symptoms, and regulating the activity of DA neurons in VTA has great potential as an antidepressant strategy (Bai et al., 2017). The mesocorticolimbic DA system originates from the VTA and mainly projects to the PFC, NAc, hippocampus, BLA, dorsal striatum, ventral striatum, and the OT, where DA transmission is partially regulated through negative feedback mechanisms via DA D2 autoreceptors located on cell bodies and terminals of VTA DAergic neurons (Gasbarri et al., 1994; Chaudhury et al., 2013) (Figure 4). The DAergic pathway from the VTA to the NAc plays a key role in reward-related phenomena and plays an important role in aberrant motivational and emotional processes involved in psychiatric disorders (Nielsen et al., 2016). DAergic neurons have been the main focus of VTA research. DAergic neurons in the VTA are key components of the reward pathway, but their activity is tightly controlled by several types of inhibitory GABAergic inputs (Polter et al., 2018) (Figure 4). VTA GABA interneurons bi-directionally regulate the activity of local DA neurons, which is the basis of reward or aversion at the behavioral level (Creed et al., 2014). The VTA contains a mixture of DAergic (∼65%) and GABAergic (∼30%) neurons, and their interactions coordinate reward-seeking behavior and influence depressive-like behavior (Margolis et al., 2006; Nair-Roberts et al., 2008; Yamaguchi et al., 2011) (Figure 4). Although DA projection neurons are usually emphasized, VTA projection neurons can express DA, glutamate, or GABA, and are capable of multiplexing combinations of these neurotransmitters as well as neuropeptides (Barker et al., 2016). VTA GABA projection neurons project to areas such as the NAc, the VP, the PFC, the LHb, the central amygdala (CeA), and the dorsal raphe nucleus (DRN), among other regions (Juarez and Han, 2016; Bouarab et al., 2019) (Figure 4). The *in vivo* discharge pattern of DA neurons in the VTA is controlled by GABA afferents mainly from the NAc and local GABA interneurons (Simmons et al., 2017). VTA GABA neurons have a variety of functions, both affecting DAergic activity through local inhibitory control and exerting DA-independent effects (Bouarab et al., 2019). For example, the optogenetic strategies of selective stimulation of VTA GABA neurons as well as their projection fibers to the NAc, suggest that the dynamic interaction between VTA DA and GABA neurons can control the initiation and termination of reward-related behaviors (van Zessen et al., 2012). In addition to the populations of VTA DA and GABA neurons, that have been studied for a long time, glutamatergic neurons have been identified in the VTA (Yamaguchi et al., 2007). Indeed, approximately 5% of the total neuronal content of the VTA) were recently discovered in the VTA, expressing the vesicular glutamate transporter 2, VGluT2 (Barbano et al., 2020). Papathanou et al. (2018) suggested that VGluT2 in mature DA neurons actively promotes glutamate neurotransmission in the NAc, and highlighted the co-release of VGluT2-mediated glutamate in the complex mechanisms of synaptic plasticity in drug addiction.

Optogenetic stimulation of the NAc lateral shell inputs to the VTA produced a robust real-time place preference and positive reinforcement of intracranial self-stimulation (Pignatelli and Bonci, 2018). Indeed, the inputs of information from the NAc subnuclei to specific VTA microcircuits will be important for a deeper understanding of the mechanisms of neuropsychiatric diseases that involve altered motivational function (Pignatelli and Bonci, 2018). The activation of D1-D2 receptor heteromers in NAc induced the enhanced expression of GABA-related proteins in NAc and VTA (Perreault et al., 2012a). Activation of the D1-D2 receptor heteromers increases GABAergic tone in the NAc and perhaps by NAc efferent inhibition of the VTA (Hasbi et al., 2017). In optogenetic experiments of DA neurons in VTA, staged optogenetic activation of these neurons can alleviate chronic stress-induced depressive-like behavior within a few seconds, a phenomenon that requires DA receptors in the NAc to function, although the specific type of receptor is not known (Zhang et al., 2019). DRD2 receptor activation enhances Kv7.4 currents through a Gi/o and redox-dependent cellular pathway, and Kv7.4 facilitates DA-induced inhibition of spontaneous firing of VTA DA neurons (Su et al., 2019). DRD2 receptor-mediated auto-inhibition may be involved in the development of depressive-like behaviors induced by stress, and thus the selective targeting of Kv7.4 is considered a promising antidepressant treatment strategy (Su et al., 2019).

### Nucleus Accumbens

The NAc is part of the striatum, which together with the olfactory tubercles makes up the ventral striatum (Marcott et al., 2018). The NAc is one of the key regions of the brain reward circuit, and in some neuropsychiatric disorders, such as depression, there is an aberrant response to rewarding and aversive signals. The NAc receives glutamatergic projections from the PFC, hippocampus, and amygdala, as well as DAergic innervation from the VTA (Groenewegen et al., 1999). As an important part of the midbrain VTA-NAc-PFC reward circuit, critical afferent projections to the NAc arise from a direct projection from the midbrain VTA (Zhou et al., 2018; Castro and Bruchas, 2019; Soares-Cunha et al., 2020). Glutamatergic synaptic transmission is mainly mediated by AMPAR and NMDAR, and the MSNs in the core and shell of the NAc receive glutamatergic input from PFC, hippocampus, and amygdala. In rodents, more than 95% of the cells in NAc are MSNs, which receive excitatory input from four major brain regions, namely the PFC, hippocampus, basolateral amygdala, and the thalamus (Sesack and Grace, 2010). Morphological evidence suggests that DA D1 and D2 receptors form complexes in the dorsal striatum and NAc of mammalian species (including mice, rats, non-human primates, and humans), and in all of these species, a higher number of MSNs expressing the D1-D2 heteromers was observed in the NAc than in the dorsal striatum (caudate and putamen) (Hasbi et al., 2020a).

**FIGURE 5:**
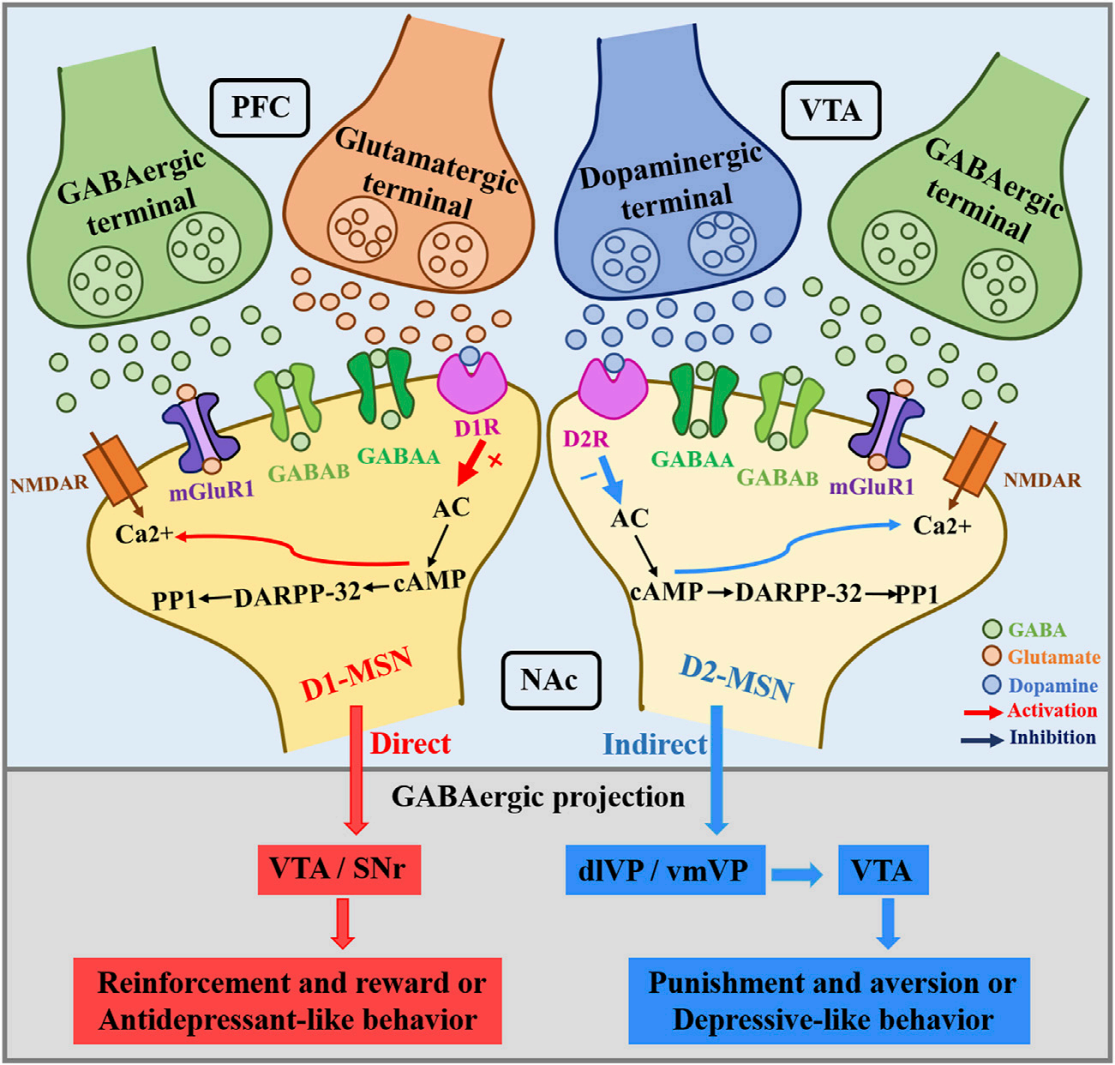
NAc dopamine receptors in D1-MSNs and D2-MSNs receive PFC glutamatergic and GABAergic inputs, and VTA dopaminergic and GABAergic inputs. The direct NAc innervation of the VTA/SNr is mainly mediated by D1-MSNs. In general, NAc core projects to dlVP, NAc shell innervates the vmVP, and this process is mainly mediated by D2-MSNs.Whereas D1-MSNs mediate reinforcement and reward or antidepressant-like behavior, D2-MSNs have been associated with punishment and aversion or depressant-like behavior. VTA, ventral tegmental area; SNr, substantia nigra pars reticulata; VP, ventral pallidum; dlVP, dorsolateral pallidum; vmVP, ventromedial pallidum.

GABA MSNs subtypes that co-express DRD1 and DRD2 also exhibit glutamatergic phenotype, thus showing a combined GABAergic/glutamatergic phenotype (Perreault et al., 2012a). The NAc mainly contains GABA-expressing MSNs divided into subtypes based on the expression of DA receptors: DRD1-containing MSNs (D1-MSNs) and DRD2-containing MSNs (D2-MSNs) (Francis et al., 2019). These two populations of MSNs constitute the main NAc output projections, which have different functional roles in stress and reward-mediated behavior (Soares-Cunha et al., 2020). Since cells expressing both receptors appear to have glutamatergic expression as well, this would suggest that this pathway acts separately from these other well-characterized GABAergic output pathways. DAergic signaling mainly acts through D1-MSNs and D2-MSNs. D1-MSNs project primarily to the VTA/SNr (the direct pathway) (Perreault et al., 2010). D2-MSNs project indirectly to the VTA/SNr *via* the dlVP/vmVP (Soares-Cunha et al., 2016; McCutcheon et al., 2019). These striatal projections are summarized in Figure 5. D1-MSNs are involved in mediating responses to reward signals, while D2-MSNs are involved in mediating responses to aversive signals (Soares-Cunha et al., 2016). The classical view of striatal D1R signaling as pre-reward/reinforcement and D2R signaling as pre-aversive is too simplistic, and it is premature to assume that neurons expressing D1R and D2R play completely independent (and opposite) roles (Soares-Cunha et al., 2016). This relationship is clearer for the dorsal striatum than for the ventral striatum, where the relationship to reward/aversion may be less distinct and dependent on the duration of stimulation (Soares-Cunha et al., 2020). The positive enhancement that is mediated by midbrain DA neurons entails the activation of D1 and D2 receptors in the NAc. Targeting D1-MSN activity may provide new therapeutic strategies for depression or other affective disorders. The mesolimbic DAergic system role in the pathophysiology of depression is more and more obvious. BDNF is elevated in the NAc of depressed patients and contributes positively to depressive-like behavior in rodents. BDNF is widely considered to be critical for neural and synaptic plasticity throughout the nervous system, and recent studies have shown that BDNF in the mesolimbic DA circuit originates from DA neurons in the VTA that project into the NAc (Koo et al., 2019). Koo et al. concluded that chronic social defeat stress (CSDS) mice exacerbate failure-induced behavioral symptoms during repeated optogenetic stimulation of the mesolimbic VTA-NAc circuit and that these behavioral symptoms can be normalized by BDNF-TrkB blockade in the NAc (Wook Koo et al., 2016). Staged stimulation of the VTA-NAc pathway promotes the release of BDNF and DA from VTA DA terminals (Bass et al., 2013). D1-D2 receptor heteromers are highly expressed in NAc and have been shown to enhance BDNF expression and signal transduction in NAc (Shen et al., 2015). Research by Rahman et al. has shown that the simultaneous activation of DA D1- and D2-like receptors in the NAc stimulates long-loop negative feedback pathway from the NAc to the VTA reducing somatodendritic DA release, while the sole activation of D1- or D2-like receptors in the NAc reduces DA terminal release but without any effect in the VTA (Rahman and McBride, 2001).

Recent research has shown that the chemokine receptor CCR2 contributes to depression associated with neuropathic pain by increasing NR2B-mediated currents in both D1- and D2- MSNs in the NAc shell (Wu et al., 2018). Further experiments showed that inhibition of CCR2 in D1R-MSN and D2R-MSN reduced SNL-induced neuropathic pain and depressive-like behavior (Wu et al., 2018). Using whole-cell patch-clamp electrophysiology, Francis et al. (2015) found that the excitatory synaptic input frequency of D1-MSNs decreased while that of D2-MSNs increased in mice that exhibited depressive-like behavior after experiencing CSDS. Notably, bidirectional alterations in D1-MSN activity promoted the opposite behavioral outcome of chronic social stress, while bidirectional modulation of D2-MSN did not alter the behavioral response to CSDS (Francis et al., 2015). The relationship of NAc neurons in rats that co-express DRD1 and DRD2, forming D1-D2 heterodimers, with depression is unclear. MSNs have been shown to have the unique property of expressing D1-D2 receptor heterodimers. The NAc exhibits relatively abundant D1–D2 heterodimers (Perreault et al., 2010), and activation of D1–D2 heterodimers in the NAc shell can alter the expression of proteins involved in GABA and glutamate activity in VTA and the SNr (Fatima et al., 2020). The polylactic acid (PLA), fluorescence resonance energy transfer (FRET), and immunoprecipitation techniques were used to establish the presence of D1-D2 heterodimers in the striatum of rats and monkeys. Perreault et al. (2010) found that in NAc cell bodies, the energy transfer between fluorescent-labeled D1R and D2R was very high, indicating a stronger receptor-receptor interaction and higher densities of heterodimers. Those authors subsequently showed that MSNs co-expressing DRD1 and DRD2 showed a unique dual GABA/glutamate phenotype and activation of the D1R–D2R heterodimers altered the expression of proteins involved in GABA and glutamate activity in regions of the mesolimbic and nigrostriatal pathways (Fatima et al., 2020). A novel mechanism that modulates depressive-like and anxiety-like behavior in rats through the DA system involves D1-D2 receptor heterodimers (Shen et al., 2015). Recent research suggests that higher D1-D2 heterodimer expression in female animals may significantly increase susceptibility to depressive-like and anxiety-like behavior (Hasbi et al., 2020c). Specifically, compared with male rats, activation of D1-D2 heterodimers in the NAc of female rats resulted in greater activation of BDNF/TrkB and Akt/GSK3/β-catenin, two important depression-related signaling pathways, and this difference may explain the greater predisposition of female rats to depressive and anxiety behaviors (Hasbi et al., 2020c). In Sprague-Dawley rats, selective activation of D1-D2 heterodimer increased grooming behavior and reduced AMPA receptor GluA1 phosphorylation via calcium/calmodulin kinase II-α, suggesting that D1-D2 heterodimer play a role in modulating the sensitivity of the reward pathway (Perreault et al., 2010). Therefore, targeting D1-MSN/D2-MSN activity may provide novel treatment strategies for depression or other affective disorders (Francis et al., 2015).

### The Prefrontal Cortex

DA regulation in the PFC plays a key role in the modulation of stress responsiveness, cognition, motivation, and emotional behavior, and DA regulation mediates a variety of effects on neuronal physiology and function in the PFC (Cohen et al., 2012). Previous studies have shown that decreased DAergic transmission in the medial PFC is associated with the pathophysiology of depression. All subtypes of D1-like receptors and D2-like receptors are present in PFC, but DRD1 receptors are the most abundant (Santana et al., 2009). The DRD1 receptor is highly expressed in the glutamatergic pyramidal cells of the PFC (Arnsten et al., 1994), while the DRD2 receptor is most commonly found in GABAergic interneurons in the PFC and plays a role in inhibiting NMDA receptor-mediated excitatory neurotransmission. In cortical regions, DA modulates excitatory postsynaptic currents (EPSCs) and inhibitory postsynaptic currents (IPSCs) through DRD1 and DRD2 receptors (Zheng et al., 1999; Trantham-Davidson et al., 2004). Activation of the D1 receptor enhances the firing of DRD1+ pyramidal cells and VIP-positive (VIP+) interneurons, which indicates that the DRD1 receptor enhances both excitatory and inhibitory microcircuits in the PFC (Anastasiades et al., 2019). Alterations in DRD1 density are associated with cognitive dysfunction in psychiatric disorders, and in the PFC DRD1 also plays a key role in the regulation of working memory (McCarthy et al., 2020). Infusions of a DRD1 DA receptor agonist directly into the mPFC and infusion of DRD2 receptor antagonist into the NAc shell, reduced stress-induced behavioral changes in DA-deficient rats, indicating that DAergic transmission via DRD1 in the mPFC modulates DRD2 -mediated stress responsiveness in the NAc (Scornaiencki et al., 2009). Pyramidal neurons (PYR), as the main output neurons in the mPFC, play an important role in stress-induced cortical dysfunction. Recent evidence has shown that PYR neurons expressing DA DRD1 (D1-PYR) or DRD2 (D2-PYR) exhibit differences in ion channel expression, inhibitory synaptic innervation, and subcortical projection targets (Anastasiades et al., 2019). DRD1 and DRD2 are expressed on glutamatergic PYR neurons in the PFC, but the role of D1 and D2 receptors expressed in PFC PYR in depression and antidepressant responses is largely unknown.

DA activates DRD1 and DRD2 receptors in PFC, signaling by stimulating Gαs or inhibiting Gαi proteins respectively, as well as β-arrestins, to regulate the activity of pyramidal neurons and interneurons (Beaulieu et al., 2007). Although several antidepressant drugs can affect the DA system of the mPFC, the role of the D1-like or D2-like receptors in the PFC region in the antidepressant process is still unclear. l-SPD, which has a unique pharmacological profile of DRD1 agonism and DRD2 antagonism exerted antidepressant-like effects on the CMS model of depression (Zhang et al., 2017). Specifically, l-SPD activates the downstream signaling of the PKA/mTOR pathway, leading to an increase in expression of the synaptogenesis-related proteins PSD 95 and synapsin I. Additionally, l-SPD also triggers long-term potentiation in the mPFC, suggesting that the D1R/PKA/mTOR signaling cascade plays a key role in l-SPD-mediated antidepressant responses (Zhang et al., 2017). Recent studies have shown that elevated mPFC DA levels may further enhance excitatory synaptic transmission through activation of the D1/PKA/DARPP32 intracellular signaling pathways, which may be the underlying mechanism of antidepressant-like effects. Recently, the mechanism of the antidepressant-like action of ketamine in the PFC region has become increasingly clear (Wohleb et al., 2017). Ketamine infusions in the ilPFC are sufficient to produce long-lasting antidepressant-like responses in rats (Fuchikami et al., 2015). Similar effects could be produced by optogenetic stimulation of ilPFC neurons, while the effects of systemic ketamine could be blocked by optogenetic inactivation. Further study demonstrated that the antidepressant effect of optogenetic stimulation was mediated by DRD1-expressing, but not DRD2-expressing mPFC neurons (Hare et al., 2019). Some of these effects were associated with structural changes in mPFC neurons. D1 receptor and its associated signaling pathways in mPFC neurons mediate acute stress-induced dendritic plasticity and contribute to the suppression of stress susceptibility (Shinohara et al., 2018).

By contrast, specific layer V pyramidal neuron subtypes in PFC selectively express DRD2, triggering post-depolarization of Ca^2+^ dependent channels, which can effectively regulate the activity of specific PFC neurons (Gee et al., 2012). The effect of the antidepressant venlafaxine on memory consolidation impairment in Wistar rats with chronic mild stress (an animal model of treatment-resistant depression) is related to the D2-like receptor inhibition in the ventromedial prefrontal cortex, suggesting an important relationship between depression and D2-like activity (Papp et al., 2019). Studies have shown that altered expression and function of DRD3 in patients and animal models of depression correlate with the severity of depression or depressive-like behavior. DRD3 has been extensively studied in animal models of LPS-induced inflammatory depressive-like behavior. LPS significantly reduces DRD3 in the VTA, mPFC, and NAc, key regions within the mesolimbic DAergic system. LPS reduces DRD3 in the VTA, mPFC, and NAc (Wang J. et al., 2018). The DRD3 agonist pramipexole had antidepressant effects in the LPS model, while the DRD3 antagonist NGB2904 induced depressive-like behavior by preventing the induction of pro-inflammatory cytokines and BDNF and ERK1/2-CREB signaling pathways. These findings provide a mechanism for the role of DRD3 in LPS-induced depressive-like behavior by mediating potential cross-effects between pro-inflammatory cytokines (tumor necrosis factor-α, interleukin-1β, and interleukin-6), BDNF, and changes in the ERK1/2-CREB signaling in the VTA and NAc. This indicates that DRD3 is a potential target for the treatment of depression.

### Hippocampus

Hp is a complex structure in the temporal lobe associated with memory, cognition, and stress. Hp has functional differences along its dorso-ventral axis reflected in differences in gene expression and anatomical connectivity (Castro-Hernández et al., 2017). The dorsal Hp (dHp) is associated with different types of memory and cognitive function, while the ventral Hp (vHp) is associated with the emotional and motivational consequences of stress, including depression and anxiety (Bagot et al., 2015). The Hp contains high levels of glucocorticoid receptors and mediates feedback inhibition to the HPA axis. Chronic activation of this system produces changes in stress responses that contribute to the development of depression. The Hp is the most frequently studied brain region in depression research, along with other areas of the brain that are associated with stress, memory formation/consolidation, and emotion, such as the PFC and amygdala (Liu et al., 2017). Numerous studies have shown that DA receptors regulate long-term synaptic plasticity and memory function in the Hp, and also play a key role in imparting novelty and reward signals that influence memory formation (Hansen and Manahan-Vaughan, 2014; Rocchetti et al., 2015; Palacios-Filardo and Mellor, 2019; Park et al., 2021). In animal models of depression, chronic and severe stress impairs Hp-dependent explicit memory formation, and this effect can be explained by changes in hippocampal synaptic plasticity, e.g., alterations in LTP and LTD (Kim and Diamond, 2002). In the dentate gyrus (DG) of the Hp, the D1-like receptor antagonists block the LTD induced by afferent stimulation (Wiescholleck and Manahan-Vaughan, 2014). Pharmacological, genetic, biochemical, and imaging methods have been used to show that activation of DRD1 in the hippocampus, but not DRD2, increases calcium inflow through NMDA receptors, which enhances the MEK-ERK and mTOR pathways (David et al., 2020). Both pathways inhibit eEF2K activity by phosphorylation of eEF2K on e366, resulting in dephosphorization of eEF2 Thr56, suggesting that eEF2 may be a promising therapeutic target for the treatment of depression (David et al., 2020). In models of desperate behavior induced by prenatal stress (PNS) and chronic unpredictable mild stress (CUMS), the selective non-ergoline DA D2-like receptor agonist ropinirole (ROPI) upregulates Hp and PFC developmental gene expression, possibly because D2 receptor agonists increase the levels of the rate-limiting enzyme TH in the Hp and PFC (Fatima et al., 2020). At the same time, downregulation of GSK-3β and enhanced BDNF and TH expression are observed, thereby promoting adult hippocampal neurogenesis and alleviating symptoms of depression (Fatima et al., 2020). In a model of LPS-induced peripheral inflammation, the DA DRD2/DRD3 agonist pramipexole (PPX) inhibited the increase in LPS-induced IL-1 expression and eliminated the increase in 3-nitrotyrosine (3-nitrotyrosine, 3-NT) in the Hp (Lieberknecht et al., 2017). However, the authors of this study also concluded that the antidepressant-like effects observed with PPX in LPS-treated mice may be dependent on its anti-inflammatory properties and may not be related to the activation of DAD2 receptors (Lieberknecht et al., 2017). Therefore, the role of dopamine D2 receptors in Hp needs to be further investigated. In rodents, the projection to the NAc from the vHp is associated with stress susceptibility, and stress-induced increases in vHp-NAc activity are consistent with the increase of spontaneous excitatory postsynaptic current frequency (Muir et al., 2020). Different subpopulations of D1+ MSNs in the NAc medial shell (NAcMS) project to the VTA(D1+^VTA^ MSNs) and the VP (D1+^VP^ MSNs) and receive inputs from the vHp and basolateral amygdala (BLA) (Baimel et al., 2019). Although the vHp and BLA inputs target D1+^VTA^ and D1+^VP^ MSNs, those vHPC inputs are stronger on D1+^VTA^ MSNs. Through optogenetic manipulation to bidirectional control of afferent-specific synaptic function, a unique role for vHp-NAc in driving depressive-like behavioral phenotypes was shown (Bagot et al., 2015). These studies suggest that circuit-level therapeutic interventions that inhibit the overactivation of presynaptic vHp may constitute an effective strategy for the treatment of depression.

### Lateral Habenular Nucleus

It has recently emerged that the LHb is a critical brain region in the pathophysiology of depression. There is growing evidence from rodent and human studies that abnormal activity in the LHb is associated with depressive symptoms, such as helplessness and lack of pleasure (Yang et al., 2018). Pharmacological and optogenetic manipulation of the LHb activity alters DAergic regulation of mPFC neuronal activity, which controls multiple brain processes that are relevant to depression. Chronic stress-induced hyperactivity VTA-projecting LHb neurons is associated with increased passive coping response, and the neurons show increased burst and tonic firing (Cerniauskas et al., 2019). Hyperactivity of LHb is found in both rodent models of depression and human patients with depression. In LHb neurons, p11 is a multifunctional protein associated with depression and is significantly upregulated under chronic restraint stress. That is, downregulation of P11 expression in LHb can reduce stress-induced depressive-like behavior (Seo et al., 2018). Moreover, overexpression of p11 in D2 receptor-containing LHb neurons induces depressive-like behaviors, suggesting that p11 in LHb may be a key molecule in the regulation of negative emotions (Seo et al., 2018). Quantitative proteomic screens show that LHb expression of the β form of calcium/calmodulin-dependent protein kinase type II (βCaMΚΙΙ) is up-regulated in animal models of depression and down-regulated by antidepressants (Li et al., 2013).

Additional evidence shows that DA function in the habenula regulates these outputs and depressive-like behavior. DRD2 has functionally important expression in LHb, especially in the middle portion of LHb, which regulates the effect of aversive stimuli on behavioral output influenced by DAergic activity (Aizawa et al., 2012). LHb DRD2+ neurons regulate emotional behavior via negative reward signals and participate in stress-induced depressive-like behavior (Matsumoto and Hikosaka, 2007; Stamatakis and Stuber, 2012). Injecting DA antagonists into mPFC blocks aversive emotions induced by activation of the LHb-VTA pathway, and increased DA neuronal activity in the mesocortical projections, either via direct LHb inputs or indirectly *via* the rostromedial tegmental nucleus (RMTg)) (Stamatakis and Stuber, 2012). Both DRD1 and DRD2 are expressed in the LHb (Chan et al., 2017). Injection of DA receptor agonists or antagonists into LHb showed that activation or inhibition of DRD1 but not DRD2 in LHb increased anxiety-like behavior, but decreased depressive-like behavior in rats (Chan et al., 2017). The above results confirm that the DRD1+ and DRD2+ LHb neurons are important molecular and cellular determinants of depressive-like behavior. DRD1 dysfunction in the LHb increases anxiety-like behavior but decreases depressive-like behavior, and impairs aversive learning in rats, suggesting that proper activation of DRD1 in LHb is important for this processing, and manipulation of LHb neurons through DRD1 may be a target for the treatment of depression (Proulx et al., 2014). Studies report that unilateral 6-hydroxydopamine lesions of the substantia nigra pars compacta (SNc) in rats induces depressive-like behavior and hyperactivity of the LHb. Intra-LHb injection of the DRD4 receptor agonist A412997 and antagonist L-741,742 increase depressive-like behavior and produce antidepressant-like effects in SNc-lesioned rats (Hui et al., 2020). These studies suggest roles for DAergic receptor in the modulation of habenula circuits involved in depression. The role of DAergic receptor heterodimers in habenula function have not yet been explored.

### Ventral Pallidum

The VP is an important node in the medial limbic network, being the primary output of the NAc and projecting to the LHb and the VTA (Wulff et al., 2019). VP is the central structure of the reward system, receiving intensive innervation from NAc, and consists mainly of GABAergic neurons, with roles in cognition and addiction (Root et al., 2015). The VP is a significant convergence point at the interface of the motivational and reward circuitry associated with depression (Smith et al., 2009). The VP mainly contains GABAergic neurons, but also contains a smaller proportion of cholinergic and glutamatergic neurons (Geisler et al., 2007; Faget et al., 2018; Tooley et al., 2018). VP neurons send projections to different areas of the brain, some associated with reward (e.g., DAergic VTA neurons) and some associated with aversion (e.g., LHb). Glutamatergic VP neurons increase the activity of neurons in the LHb, medial tegmental nucleus, and GABAergic VTA neurons and adaptively limit reward-seeking (Tooley et al., 2018). It has been shown that the VP is the convergence point of MSN expressing DA receptor type 1 (D1-MSNs) and type 2 (D2-MSNs) of the NAc (Creed et al., 2016), and targeting VP may provide a new therapeutic strategy for depression. There are two discrete circuits of parvalbumin-positive (PV) neurons in the VP, which project to either the LHb or the VTA, with consequently different potential roles in the pathogenesis of depression (Knowland et al., 2017). Optogenetic techniques have revealed that both excitatory and inhibitory VP cells drive motivational behavior, and fine-tuning these inhibitory/excitatory signaling pathways is critical for normal hedonic and motivational processes (Faget et al., 2018).

## Potential Antidepressant-Like Effects of Dopamine Receptor Agonists/Antagonists

The role of DA receptors in depression has attracted increasing attention recently. DA receptors have been identified in many brain regions associated with the development of depression. Unfortunately, traditional approaches that directly manipulate DA receptors cannot be used in clinical practice because of their effects on blood pressure (Sassano-Higgins et al., 2011; Wang S. et al., 2018). An increasing number of studies have shown that the action of DA receptor modulators may be a potential treatment for depression if these peripheral DA effects can be overcome. In recent years, PET imaging with [11C]-(+)-PHNO has enabled researchers to assess *in vivo* the occupancy of DA receptors and/or their down- or up-regulation by given drug treatment (Leggio et al., 2016). Today, the search for useful molecular determinants of DA selectivity seems achievable. The advances in D1- and D2-like receptor agonists and antagonists have provided more selective compounds that may be able to selectively target different DA receptors and perhaps address depression-related symptoms. D1R is a promising drug target, where its selective activation may provide a new approach for the treatment of depression. In female rats, repeated injections of the D1 receptor partial agonists SKF 83959 increase BDNF expression and TrkB activation, thereby affecting depressive and anxiety-like behavior (Hasbi et al., 2020c). Pergolide targets DRD1 and DRD2 receptors and improves visual-spatial working memory, verbal learning and memory, and executive function in schizotypal patients (McClure et al., 2010), indicating that DA agonists may be beneficial for cognitive abnormalities in schizophrenia spectrum disorders. These effects may extend to antidepressant effects. Studies have shown that depressive-like behavior induced by chronic unpredictable stress (CUS) is accompanied by a significant decrease in both DA levels and DRD2 expression in NAc. Before CUS in Sprague Dawley rats, infusion of the DRD2 agonist Quinpirole and DRD2 antagonist eticlopride to NAc reversed depressive-like behavior induced by CUS and normalized DA levels in NAc (Qiao et al., 2020). Interestingly, the most recent studies found that Partial agonist activity of the DRD2 is a key feature of third-generation antipsychotics (TGAs), which have antidepressant-like effects and improved cognitive performance (Chen et al., 2022). The unique and selective DRD2-selective partial agonist (−)-IHCH7041 may provide the medical community with chemical tools for exploring signaling pathways that counteract the efficacy and side effects of psychiatric disorders such as depression (Chen et al., 2022). A growing body of evidence also suggests that DRD3 receptor antagonists may be effective antidepressants (Maramai et al., 2016). Cariprazine is a partial agonist of D2/D3 receptors that has recently been approved in the United States for the treatment of psychiatric disorders (Findlay et al., 2017). Cariprazine reduces anhedonia resulting from chronic unpredictable stress and shows an effective antidepressant effect comparable to aripiprazole and the tricyclic antidepressant imipramine (Duric et al., 2017). Furthermore, the antianhedonic-like effect of Cariprazine was not observed in D3 knockout mice, suggesting that the cariprazine antidepressant-like activity is mediated by DRD3 (Duric et al., 2017). Aripiprazole, Blonanserin, and the D2/D3 receptors partial agonist Cariprazine play an important role in the treatment of depression, and this may not be possible without the role of D3R (Leggio et al., 2016). Therefore, the development of novel, more selective chemical scaffolds for D3R ligands may be essential. Injection of the DRD4 agonist A-412,997 or the antagonist L-741,742 into the LHb affects the expression of depressive-like behaviors and produces antidepressant-like effects in SNc-lesioned rats (Hui et al., 2020). In general, DA receptor agonists/antagonists will be a new option for the treatment of depression. Representative DA receptor agonists and antagonists and their role in the treatment of depression are summarized in Table 2.

**TABLE 2:** Representative dopamine receptor agonists and antagonists and their role in the treatment of depression.

Dopamine receptor agonist	Representative substance	Subjects (Methodology)	Effects on depressive-like behavior	References
D1R Agonist	Pergolide	41 non-demented patients suffering from mild or moderate depression and Parkinson’s disease	Demonstrated antidepressant effects in PD patients	Rektorová et al. (2013)
	SKF-81297	Chronically stressed rats	100 ng of SKF 81297 significantly ameliorate depressive-like behavior	Mizoguchi et al. (2002)
	SKF-38393	Adult ovariectomized female rats	Blocked the antidepressant-like effect	Fedotova and Ordyan (2011)
D2R Agonist	Cabergoline	Male Wistar and Wistar-Kyoto rats	Antidepressant-like property	Chiba et al. (2010)
	Piribedil	The placebo-controlled, randomized, double-blinded trial was conducted in 37 patients with Parkinson’s disease presenting with apathy	Dopamine agonist piribedil improves apathy in Parkinson’s disease	Thobois et al. (2013)
	Quinpirole	Sprague Dawley rats	Delivery of quinpirole into the NAc of control animals induced depressive-like behaviors	Qiao et al. (2020)
	Pramipexole	five RCTs, three open-label trials, and five observational studies, with 504 participants	Patients treated with Pramipexole showed improvement in depressive symptoms	Tundo et al. (2019)
	Ropinirole	32 unipolar and bipolar patients who remained depressed	No difference in primary or secondary outcome measures was detected between the treatment and control groups	Gershon et al. (2019)
	L-742,626	Wistar or Wistar-Kyoto rats	Venlafaxine reversed the effect of L-742,626 in controls	Papp et al. (2019)
D3R Agonist	Rotigotine	48 PD patients	Rotigotine improves apathy, depression, and anxiety in PD patients	Castrioto et al. (2020)
D4R Agonist	PD-168,077	174 transverse hippocampal slices (400 μm) prepared from 87 male Wistar rats	D4R activation induces synaptic depression	Navakkode et al. (2017)
D1/D5R Agonist	SKF-38393	Adult zebrafish (AB wild-type; ∼50:50 male: female ratio at 4-month of age)	Pretreatment with the agonist SKF-38393 protects subjects from conspecific alarm substance (CAS, a natural stressor)	Fontana et al. (2021)
D1R Antagonist	SKF-83566	Forty-nine male Long–Evans rats	Administration of SKF 83566 blocked LTP in mPFC and resulted in long-term depression induced by high-frequency stimulation	Coppa-Hopman et al. (2009)
	Haloperidol	seven patients (five men and two women; mean age = 36.7 years, SD = 13.8, range = 26–61)	The first description of the efficacy and safety of the SSRI citalopram in combination with haloperidol in the treatment of psychotic depression	Bonomo et al. (2002)
D2R Antagonist	Raclopride	Adult outpatients with depression >59 years old	Depression status is associated with lower [[11C] raclopride binding	Rutherford et al. (2019)
	Sulpiride	ten patients and ten age-matched male volunteers	Improve depressive-like behavior and may have the effect of increasing dopamine turnover	Verbeeck et al. (2001)
	Risperidone	16-week randomized placebo-controlled trial for participants concurrently treated with risperidone	A combination of Risperidone and Omega-3 improves depressive-like behavior	Robinson et al. (2019)
D3R Antagonist	7-OH-DPAT	Wistar or Wistar-Kyoto rats	Potential anti-anxiety and antidepressant effects	Rogóz et al. (2004)
D4R Antagonist	L-741,742	Male Sprague Dawley rats	Electrophysiological currents were inhibited by DA-D4-receptor antagonist L-741,742 and it was observed in LHb neurons when DA uptake or degradation was blocked	Root et al. (2015)
D1/D5R Antagonist	SCH-23390	Gerbil (*Meriones unguiculatus*, *n* = 130) pups	Decreasing dopamine receptor signaling diminishes social learning	Paraouty et al. (2021)

## Conclusion

According to the types of DA receptors and their distribution in different brain regions, this paper reviews the current research status of the molecular, cellular and neural circuit mechanisms of DA receptors involved in depression, including the research progress into the role of DA receptor D1-D2 heterodimers. Understanding the function and localization of DA and its receptors in the brain and the complexity of their signaling mechanisms as well as pharmacological strategies based on receptor complexes may have potential new applications in the depression pathogenesis. Multidimensional analysis of DA receptors and DA receptor-related mechanisms or post-receptor signaling cascades will provide an exciting opportunity for depression treatment, which will minimize the side effects of depression, and these approaches may be closely related to the metabolic targeting of DA receptors and heterodimers and their downstream intracellular signaling events.

## Glossary

5-HT: serotonin
A2ARs: adenosine A2A receptors
AADC: aromatic L-amino acid decarboxylase
AC: adenylate cyclase
ACC: anterior cingulate cortex
Akt: protein kinase B
AMPAR: AMPA-type glutamate receptors
BDNF: brain-derived neurotrophic factor
BLA: basolateral amygdala
CamKII: Ca^2+^/calmodulin-dependent protein kinase II
cAMP: cyclic adenosine monophosphate
CNS: central nervous system
COMT: cathecholamine O methyltransferase
CP-AMPAR: Ca^2+^ -permeable AMPA receptor
CREB: cAMP-response element binding protein
CSDS: chronic social defeat stress
CSF: cerebrospinal fluid
CUMS: chronic unpredictable mild stress
D1/2/3/4/5R: dopamine receptor subtype 1/2/3/4/5
D1-MSNs: DRD1-containing MSNs
D2-MSNs: DRD2-containing MSNs
DA: dopamine
DAergic: dopaminergic
DARPP-32: dopamine- and cAMP-regulated phosphoprotein
DAT: DA transporter
DBS: deep brain stimulation
DG: dentate gyrus
dlVP: dorsolateral subcompartment of the ventral pallidum
DRD1: dopamine receptor D1
DRD2: dopamine receptor D2
DRD3: dopamine receptor D3
DRD4: dopamine receptor D4
DRD5: dopamine receptor D5
ECT: electroconvulsive treatment
EPSCs: excitatory postsynaptic currents
ERK: extracellular signal-regulated kinase
fMRI: functional magnetic resonance imaging
FRET: fluorescence resonance energy transfer
GABA: gamma-aminobutyric acid
GABR_A6_: GABA receptor alpha 6 subunits
GPCR: G protein-coupled receptor: G protein-coupled receptors
GPCR: G protein-coupled receptor: G protein-coupled receptors
GSK-3: glycogen synthase kinase 3
HVA: homovanillic acid
ilPFC: inferolateral prefrontal cortex
IP3-R: inositol triphosphate receptors
IPSCs: inhibitory postsynaptic currents
JNK: c-Jun N-terminal kinase
l-DOPA: L-3,4-dihydroxyphenyl-L-alanine
LHb: lateral habenular nucleus
LNAA: large neutral amino-acid
LPS: lipopolysaccharide
LTD: Long-term depression
LTP: Long-term potentiation
MAO: monoamine oxidase
MAPK: mitogen-activated protein kinases
MDD: major depressive disorder
mPFC: medial prefrontal cortex
MSNs: medium spiny neurons
NAc: nucleus accumbens\
NaSSA: noradrenergic and specific serotonergic antidepressants
NE: norepinephrine
NMDAR: NMDA-type glutamate receptors
OCT3: organic cation transporter 3
PET: Positron emission tomography
PFC: prefrontal cortex
PH: phenylalanine hydroxylase
PKA: protein kinase A
PLA: proximity ligation assay
PLC: phospholipase C
SNr: substantia nigra reticulata
SNRIs: dual 5-HT and NE reuptake inhibitors
SSRIs: selective 5-HT reuptake inhibitors
TCAs: tricyclic antidepressants
TH: tyrosine hydroxylase
TrkB: tropomysin related kinase B
VMAT: vesicular monoamine transporters
vmVP: ventral medial of the ventral pallidum
VP: ventral pallidum
VTA: ventral tegmental area
